# Effect of initiation of invasive ventilation in acute hypoxemic respiratory failure using sequential trials: treatment efficacy instead of timing

**DOI:** 10.1186/s13054-025-05464-x

**Published:** 2025-06-03

**Authors:** Carmen A. T. Reep, Leo Heunks, Evert-Jan Wils

**Affiliations:** 1 Department of Intensive Care, Erasmus Medical Center, Rotterdam, The Netherlands; 2 Department of Intensive Care, Radboud University Medical Center, Nijmegen, The Netherlands; 3 Department of Intensive Care, Franciscus Gasthuis & Vlietland, Rotterdam, The Netherlands

In *Critical Care*, Mellado-Artigas and colleagues [1] aimed to evaluate the effect of initiating invasive mechanical ventilation (IMV) immediately versus waiting in patients with hypoxemic respiratory failure on one-year mortality. They used the target trial emulation framework, which is an increasingly recognized approach for designing observational studies that aim to compare treatment strategies [2]. Specifically, the authors used a sequential trials approach [3, 4], in which eligibility for initiating IMV is reassessed hourly during the first 48 h following the onset of hypoxemia. At each hour, a new trial is emulated using all patients eligible at that time point, and all 48 trials are combined into a single analysis. This method efficiently leverages all available observational data. However, it does not address whether immediate initiation of IMV reduces the hazard of dying compared to waiting, as implied by the title and research question. For example, a patient who remains eligible for IMV for 30 h before initiation cannot reasonably be considered as starting IMV “immediately.” The sequential trials approach instead evaluates treatment efficacy, in this case that initiating IMV reduces the risk of death compared to not initiating it at all. The authors’ conclusion does align with this efficacy question. However, the intended question of optimal timing for IMV initiation remains unanswered and highly relevant in clinical practice. While this can still be investigated within the target trial emulation framework, it would require a slightly different analytical approach. We outline three such alternatives below.

One option would be to stick to a clinical threshold at which IMV initiation is hypothesized to be beneficial, such as S/F < 200 (in line with the original paper). Then comparing two treatment strategies: early initiation (e.g., within 1 h of meeting these criteria) with delayed initiation (after 1 h). But now, instead of including patients each time they meet the criteria, only the first instance of meeting these criteria should be included. By moving away from the sequential trials approach, this method does directly address the question of whether immediate initiation of IMV reduces the risk of mortality.

A second option is to define early versus delayed initiation based on the severity of the patient’s illness. For instance, initiation during the ‘less severe stage of hypoxemia’ (e.g., S/F < 200) could be considered early, while initiation during ‘more severe stages’ (e.g., S/F < 100) could be considered delayed. Different treatment strategies could then be compared, each defined as ‘initiate IMV once the S/F ratio falls below a specific threshold,’ all using the same eligibility criteria as the start of follow-up. These strategies, in which treatment decisions depend on evolving patient characteristics, are known as dynamic treatment regimes [5]. This approach also allows for the inclusion of more than two treatment strategies, which is particularly useful when the optimal threshold for initiating IMV is uncertain. For example, Yarnell and colleagues [6] applied such an approach using S/F ratio thresholds of 88, 98, and 110.

In line with defining early versus delayed initiation based on severity of illness, a third option would be to keep the sequential trials approach but stratify patients by severity of illness at baseline, for example using different S/F ratio ranges. This would help determine whether initiating IMV is beneficial across all stages of hypoxemia, or whether the benefit is less evident in patients with milder hypoxemia (e.g., for those with an S/F ratio between 150 and 200), which could support a more delayed initiation strategy.

In conclusion, Mellado and colleagues address a clinically relevant question using an innovative methodological framework. However, the analytical approach they apply does not align with the question they aim to answer. We propose three alternative analytical approaches that more directly address the issue of timing of IMV initiation. Of note, these approaches may also be applicable to other timing-dependent treatment questions in critical care.

## Data Availability

No datasets were generated or analysed during the current study.

## Abbreviations

IMV: Invasive mechanical ventilation
S/F: SpO_2_/FiO_2_
